# Assessing the reliability of the short form 12 (SF-12) health survey in adults with mental health conditions: a report from the wellness incentive and navigation (WIN) study

**DOI:** 10.1186/s12955-018-0858-2

**Published:** 2018-02-13

**Authors:** Tianyao Huo, Yi Guo, Elizabeth Shenkman, Keith Muller

**Affiliations:** Department of Health Outcomes and Biomedical Informatics, University of Florida, P.O. Box 100177, Gainesville, FL 32610 USA

## Abstract

**Background:**

Although Short Form (SF)-12 × 2® has been extensively studied and used as a valid measure of health-related quality of life in a variety of population groups, no systematic studies have described the reliability of the measure in patients with behavioral conditions or serious mental illness (SMI).

**Methods and results:**

We assessed the internal consistency, split-half reliability and annual test-retest correlations in a sample of 1587 participants with either a combination of physical and behavioral conditions or SMI. The Mosier’s alpha was 0.70 for the Physical Composite Scale (PCS) and 0.69 for the Mental Health Composite Scale (MCS), indicating good internal consistency. We observed strong correlations between physical functioning, physical role and body pain scales (r = 0.55–0.56), and between social functioning, emotional role, and mental health (r = 0.53–0.58). We calculated split-half reliabilities to be 0.74 for physical functioning, 0.75 for physical role, 0.73 for emotional role and 0.65 for mental health respectively. We assessed the annual test-retest correlation using intraclass correlation (ICC) and found an ICC of 0.61 for PCS and 0.57 for MCS composite scores, adjusting for age, sex, race/ethnicity, and CRG. We found no decline in the correlations between baseline and the following study years until year 3.

**Conclusions:**

Our results encourage using SF-12v2® to assess health-related quality of life in the Medicaid population with combined physical and behavioral conditions or similar cohorts.

**Trial registration:**

The WIN study was registered with clinicaltrials.gov on April 22, 2015. Trial registration number: NCT02440906. Retrospectively registered.

## Background

Behavioral health conditions affect as many as 25% of the adults in the United States, particularly in individuals with low incomes. Behavioral conditions have also been shown to be associated with increasing occurrence of chronic diseases such as cardiovascular diseases, diabetes, obesity, asthma, epilepsy and cancer Patients with comorbid physical and mental conditions were historically understudied, and these patients often suffer in poor quality of life and their health care are poorly managed [1–3].

Medicaid is the largest payer for behavioral conditions and serious mental illness. Based on the *Report to Congress on Medicaid and CHIP*, about half of non-dually eligible Medicaid enrollees under age 65 with the disability had a behavioral health diagnosis in 2011 and their medical expenditures account for two-thirds of total Medicaid spending. STAR+PLUS is a Texas Medicaid program providing both the Medicaid health care and long-term services and support, through participating managed care plans. The primary goal of the STAR+PLUS program is to improve quality of care for individuals with disabilities through coordinated and comprehensive care in a cost-efficient way. Details about STAR+PLUS program has been described elsewhere [4, 5]. A reliable health-related quality of life (HRQOL) measure is critical for us to assess the well-being of this population and to quantify the efficacy of interventions to further improve the quality of care and reduce medical costs for this chronically ill population.

As one of the ten projects funded under Section 4108 of the Affordable Care Act through the Centers for Medicare and Medicaid Services, the Wellness Incentives and Navigation (WIN) project was conducted in collaboration with the Texas Medicaid Program. The State Medicaid Program desire to use an HRQOL instrument that was brief, tested through the WIN Project and had the potential to be incorporated into the STAR+PLUS Program following study completion. The Optum™ Short Form SF-12v2® instrument was then selected to provide participant reported information about physical and mental HRQOL.

The SF-12 is one of the most widely used instruments for assessing self-reported HRQOL. Originally developed from the Medical Outcomes Study (MOS) 36-item Short-Form Health Survey SF-36 [6], the SF-12v2® covers the same eight health domains as the SF-36 with substantially fewer questions, making it a more practical research tool, especially among populations with limited attention spans or mental health problems. The reliability of the SF-36 has been documented among various populations. For example, studies have reported good reliability of SF-36 in patients with schizophrenia [7] and bipolar disorder [8]. However, few studies have investigated the reliability of SF-12, including both SF-12 versions 1 (SF-12v1) and 2 (SF-12v2) among populations with mental health conditions [9]. Given the potential of SF-12v2® to measure HRQOL among populations with mental health conditions, it is important to assess the reliability of SF-12v2®.

We assessed the reliability of SF-12v2® among 1587 individuals with behavioral or serious mental health conditions enrolled in the Texas STAR+PLUS Medicaid Managed Care program who also participated in the Wellness Incentive and Navigation (WIN) project. [10]. We report the internal consistency, split-half reliability, and long-term (annual) test-retest correlations of the SF-12 instrument. Our study expands the current literature on psychometric properties of the SF-12 instrument and provides important information for planning future studies using this instrument.

## Methods

### Study cohort

The WIN project is a three-year longitudinal randomized pragmatic clinical trial funded by the Center for Medicare & Medicaid Services’ Medicaid Incentives for the Prevention of Chronic Conditions portfolio [4]. The WIN project examines the comparative effectiveness of personal navigators, motivational interviewing (MI), and a flexible wellness expense account on health care costs, cardiovascular risk factors, physical health, and HRQOL among individuals in Medicaid with co-occurring physical and mental health conditions or serious mental illness (SMI) or both, relative to usual care provided by a specialized Medicaid Managed Care Program for individuals with disability. The design of the WIN study has been described elsewhere [5]. In Brief, we recruited a total of 1663 participants in the study. We randomized participants in the Harris (Houston, Texas) service delivery area (SDA) to either a control group (*n* = 630) who received regular Medicaid managed care or an intervention group (*n* = 629) with personal navigators and a flexible wellness expense account. The Harris SDA was selected because it is where the STAR+PLUS program began, with sufficient infrastructure, experience, and stability to conduct a pragmatic clinical trial.

In order to evaluate the presence or lack of Hawthorne effect [11, 12], as well as to increase generalizability based on the comparison between the control and comparison groups, a random sample of 404 enrollees in STAR+PLUS Medicaid Managed Care program residing were recruited from the Nueces and Bexar service areas instead of the Harris service area as a comparison group. The comparison group met the same criteria as the control and intervention groups except for the location of the participants.

Among the recruited participants, 1587 of them had complete data on all twelve items of the SF-12 questionnaire that are required for computing the inter-item correlations. We only included the control group in the longitudinal test-retest analysis since the intervention may affect SF-12 scores. The accumulative loss-to-follow-up rate was 12% at the end of study year 1, 17% for year 2 and 24% for year 3. In this assessment of the reliability of SF-12 health survey, we pooled the baseline data in intervention, control, and comparison group to yield larger and more heterogeneous sample to improve the generalizability of the results.

### Inclusion and exclusion criteria

Since WIN project is a pragmatic trial, our goal is to provide evidence for adopting the intervention to the real world Medicaid population with mental or co-occurred physical and behavioral conditions. The detailed diagnostic criteria with detailed ICD-9 codes for all included/excluded co-morbidities for individuals in the WIN study was published previously [4]. In brief, eligibility for the WIN trial included the presence of a serious mental illness (SMI) diagnosis (e.g. schizophrenia, bipolar disorder, major depressive disorder) or a behavioral health diagnosis (e.g., anxiety, depression, substance use disorder) coupled with a chronic physical health diagnosis (e.g. diabetes, Chronic Obstructive Pulmonary Disease (COPD)) or a combination of all three, of sufficient severity that the individual was disabled and receiving supplemental security income. We used Medicaid enrollment files linked to health care claims and encounter data to identify individuals meeting the eligibility criteria, and contacted them by letter and phone. We excluded members with a diagnosis of dementia, Alzheimer’s disease, or intellectual disability due to concerns about impairment or limitations in understanding the program benefits. We did not collect medical treatment information from electronic health records from the participants. All participants provided verbal consent prior to participation.

### Instrument

The SF-12v2 is a health-related quality-of-life questionnaire consisting of twelve questions that measure eight health domains to assess physical and mental health. Physical health-related domains include General Health (GH), Physical Functioning (PF), Role Physical (RP), and Body Pain (BP). Mental health-related scales include Vitality (VT), Social Functioning (SF), Role Emotional (RE), and Mental Health (MH). The instrument has been validated across a number of chronic diseases and conditions [9, 13–16]. We administered the SF-12v2® annually by telephone survey to WIN study participants for three years. For each participant, we then calculated two summary scores of the SF-12v2®—physical and mental health—using the weighted means of the eight domains.

### Statistical methods

The power and sample size calculation for the WIN study was reported previously [5]. We did a post hoc power analysis to ensure we have sufficient samples to assess the test-retest correlation of the instrument within a year. With 417 subjects, we had 94% power to detect a Pearson’s correlation coefficient of 0.7 when the correlation coefficient under the null hypothesis is 0.60 using a two-sided test with the alpha level of 0.05.

We reported baseline demographics as mean ± SD for continuous variables or n (%) for categorical variables. We followed the method described in the SF12v2® manual to compute the score for each domain as well as the physical and mental composite scores [17]. Before conducting correlation analyses, we computed residuals for all eight scales using general linear model adjusting for age, gender (male versus female), race/ethnicity (white, black and Hispanic) and clinical risk groups (CRGs). The 3 M CRG is a classification system that uses standard claims data to group individuals into one of 9 health status categories, from healthy to catastrophic conditions [18]. Given that the population all had one or more chronic conditions, the CRG categories were collapsed into three chronic condition categories by combining category 1–4 as the minor, category 5 as the moderate and category 6–9 as severe chronic conditions. We compared the CRG status between race/ethnicity categories to assess whether the overall health status of the participants differs among racial/ethnic groups. We assessed internal consistency of physical and mental composite scores (PCS and MCS) using Mosier’s formula [19] as well as Pearson’s correlations between the eight scales in all patients. For the scales measured by two items, we tested split-half reliability using ICCs followed by the Spearman-Brown correction in all the respondents [20].

The original purpose of the WIN study was not to measure test-retest reliability, but to assess the effectiveness of the intervention. We conducted the retests of SF-12 annually instead of weekly or monthly across three study years, which allows us to observe the long-term decay in the reliability of SF-12 in the WIN population between any of the four time points. We used correlations among three years to assess the longitudinal decay in the reliability of SF-12 in the control group for all the scales as well as the composite scores. For each scale, we also computed the ICCs for the four repeated assessments at baseline, year 1, 2 and 3, using a mixed model (PROC MIXED) with REML estimation and Kenward-Roger approximation, adjusting for age, sex (male vs. female), race/ethnicity (Hispanic, non-Hispanic white, non-Hispanic black), and CRG. We conducted all analyses using SAS version 9.4 (SAS Institute, Cary, NC), which is considered statistically significant when *P*-value ≤0.05.

## Results

### Participant characteristics

Table 1 presents demographic characteristics of the 1587 participants. The mean age of the participants are 44 ± 9 years (range: 22–56 years old), with 64% of females, 28% of Hispanics, 38% of non-Hispanic white and 34% of non-Hispanic black. The three study groups shared similar characteristics except for a much higher proportion of Hispanics in the comparison group (60%) compared to the intervention (18%) and control groups (20%). The participants in comparison group reside in Corpus Christi, San Antonio and the immediate surrounding counties, which comprise the Nueces and Bexar service areas. Based on US census data on April 1st, 2010, 40.8% of the population are Hispanic or Latino in Harris County, where the control and comparison group were recruited. In contrast, 60.6% and 58.7% of the populations are Hispanic or Latino in Nueces and Bexar Counties, respectively, where the comparison group was recruited. This explains that higher percentage of Hispanic in the comparison group. We found no statistically significant difference in SF-12 PCS and MCS scores between control, intervention and comparison groups at baseline, suggesting the difference in the demographic profile between the comparison group and the other two study groups did not significantly affect the baseline SF-12 scores. We computed CRG status by race/ethnicity in the WIN population at baseline. The CRG status was 4% minor, 21% moderate and 75% severe chronic conditions in Hispanic participants, similar to the percentages in blacks (4% minor, 19% moderate and 77% severe) and whites (6% minor, 17% moderate and 77% severe). No statistically significant difference was observed in CRG status among racial/ethnical groups.

**Table 1:** Demographic Characteristics by Intervention Groups

Characteristics	Total	Intervention	Control	Comparison	
	n = 1587	*n* = 617	n = 617	*n* = 353	P-value
Age (years)	44 ± 9	45 ± 9	44 ± 9	45 ± 9	0.11
Gender					
Male (%)	566 (35.7)	235 (38.1)	239 (38.7)	92 (26.1)	< 0.0001
Female (%)	1021 (64.3)	382 (61.9)	378 (61.3)	261 (73.9)	
Ethno-Racial Group					
Non-Hispanic White	605 (38.2)	228 (37.0)	267 (43.3)	110 (31.2)	< 0.0001
Non-Hispanic Black	541 (34.1)	280 (45.4)	228 (37.0)	33 (9.4)	
Hispanics	441 (27.8)	109 (17.7)	122 (19.8)	210 (59.5)	
Clinical Risk Group					
Minor chronic conditions	77 (4.9)	26 (4.2)	36 (5.8)	15 (4.3)	0.34
Moderate chronic conditions	294 (18.5)	118 (19.1)	120 (19.5)	56 (15.9)	
Major chronic conditions	1216 (76.6)	473 (76.7)	461 (74.7)	262 (79.9)	
Education Level					
8th grade or less	152 (9.6)	37 (6.0)	59 (9.6)	56 (15.9)	< 0.0001
Some high school	444 (28.0)	143 (23.2)	191 (31.0)	110 (31.2)	
High school graduate, GED	547 (34.5)	215 (34.9)	217 (35.2)	115 (32.6)	
Some college or 2 year degree or more	373 (23.5)	152 (24.6)	150 (24.3)	71 (20.1)	
Unknown	71 (4.5)	70 (11.4)	0 (0)	1 (0.3)	
Diagnosis Group					
SMI	544 (34.3)	209 (33.9)	218 (35.4)	117 (33.1)	0.003
BH + PH	483 (30.4)	200 (32.4)	200 (32.4)	83 (23.5)	
SMI + BH + PH	560 (35.3)	208 (33.7)	199 (32.3)	153 (43.3)	

Table 2 show PCS, MCS, and the eight scales of the participants at baseline. The tables reflect significantly lower scores than the mean score 50, which was the average value in the 1998 US population (*p* < 0.0001). Summary scores and scores for the individual scales remained stable across time, with average PCS scores of 34.5–35.0 across three years without any obvious trend. We observed a similar pattern for MCS scores, which range from 37.5–38.5.

**Table 2:** SF-12 T Scores of Individual Scales at Baseline

.		All (n = 1587)		Intervention (n = 617)		Control (n = 617)		Comparison (n = 353)		P-value
		Mean	(SD)	Mean	(SD)	Mean	(SD)	Mean	(SD)	
Physical	PF	34.3	(12.1)	34.8	(12.3)	34.4	(12.2)	33.0	(11.7)	0.07
	RP	34.5	(10.3)	34.1	(10.3)	35.2	(10.2)	33.9	(10.4)	0.06
	BP	33.0	(14.1)	33.9	(13.8)	33.5	(14.4)	30.7	(14.0)	0.002
	GH	31.2	(11.3)	31.2	(10.9)	32.1	(11.9)	29.5	(10.9)	0.003
Mental	VT	41.4	(11.1)	41.1	(11.1)	42.2	(11.1)	40.8	(11.3)	0.10
	SF	32.8	(13.2)	33.5	(13.4)	33.5	(13.4)	30.6	(12.5)	0.001
	RE	32.3	(12.8)	32.1	(12.5)	32.9	(12.8)	31.7	(13.4)	0.32
	MH	36.8	(12.3)	37.1	(12.3)	37.5	(12.2)	35.2	(12.4)	0.01
Summary	PCS	34.0	(11.3)	34.3	(11.1)	34.5	(11.5)	32.6	(11.3)	0.03
	MCS	36.8	(12.5)	36.8	(12.6)	37.5	(12.5)	35.5	(12.4)	0.07

### Correlation between summary scores and individual scales

We calculated the Mosier’s alpha to be 0.70 for the PCS and 0.69 for the MCS, indicating strong internal consistency. Table 3 presents the correlations between the PCS and MCS summary scores and eight individual scales. We calculated similarly high correlation coefficients (r = 0.55–0.56) between PF, RP, and BP. The correlation between GH and the other three physical related scales (PF, RP, and BP) was only 0.36–0.42. The high correlations between physical health-related scales are consistent with strong Mosier’s alpha for PCS. In addition, we observed a modestly high correlation between SF, RE, and MH (r = 0.53–0.58), but a relatively lower correlation between VT and the other three mental health-related scales such as  RE (r = 0.35).

**Table 3:** Pearson’s Correlation Coefficients between Scales and Summary Scores of SF-12 in WIN Participants (n = 1587)

Variable	PCS	MCS	PF	RP	BP	GH	VT	SF	RE	MH
PCS	1.00									
MCS	−.08 (.05)^a^	1.00								
PF	.82 (.89)	.08 (.19)	1.00							
RP	.70 (.86)	.28 (.33)	.56 (.79)	1.00						
BP	.77 (.80)	.24 (.33)	.55 (.66)	.55 (.73)	1.00					
GH	.58 (.68)	.26 (.36)	.38 (.57)	.36 (.57)	.42 (.53)	1.00				
VT	.35 (.42)	.56 (.63)	.32 (.43)	.37 (.47)	.39 (.46)	.41 (.48)	1.00			
SF	.29 (.47)	.70 (.70)	.34 (.50)	.42 (.60)	.43 (.56)	.31 (.48)	.42 (.48)	1.00		
RE	.16 (.34)	.78 (.79)	.31 (.47)	.49 (.58)	.37 (.50)	.28 (.44)	.35 (.43)	.53 (.65)	1.00	
MH	.08 (.17)	.86 (.91)	.25 (.31)	.33 (.59)	.35 (.42)	.31 (.40)	.49 (.56)	.56 (.60)	.58 (.73)	1.00

^a^ The number outside the parenthesis is the correlation coefficients for measures in WIN participants. The number in the parenthesis presents correlations for the 1998 US general population [10]

### Split-half reliability

In the assessment of split-half reliability (*n* = 1587), we found the PF, RP, and RE scales all showed high split-half reliability of 0.74, 0.75 and 0.73 respectively. We determined the split-half reliability of the MH scale to be 0.65, which was slightly lower than the other three scales.

### Test-retest correlation

Table 4 show PCS, MCS, and the eight scales of the participants across all three years, and Table 5 reports test-retest correlations of the two summary scores and individual scales in SF-12v2® in the control group (*n* = 417). The average correlation coefficients for PCS and MCS between two consecutive years of 0.71 and 0.60 respectively. PCS and MCS summary scores indicated higher test-retest correlations than the individual scales. We recorded higher test-retest correlations in the physical health-related scales than the mental health-related scales. Across all eight scales, a minimal decay occurred in the correlation between baseline to year 1 and baseline to year 2. However, the correlation between baseline and year 3 is much lower than that in previous years. For instance, we calculated a correlation coefficient of PF between baseline and year 1 and 2 at 0.63 and 0.66 respectively, which dropped to 0.57 in year 3. We calculated half-widths of 95% confidence intervals for the correlation coefficients in the range of ±0.04 to ±0.07. We found an ICC of 0.61 for PCS and 0.57 for MCS, adjusting for age, sex, race/ethnicity, and CRG.

**Table 4:** SF-12 T Scores of Individual Scales in Control Group by Year

		Baseline (n = 617)		Year 1 (*n* = 530)		Year 2 (*n* = 493)		Year 3 (*n* = 491)		P-value
		Mean	(SD)	Mean	(SD)	Mean	(SD)	Mean	(SD)	
Physical	PF	34.4	(12.2)	35.2	(12.3)	35.1	(12.7)	34.8	(11.8)	0.19
	RP	35.2	(10.2)	35.9	(10.2)	35.7	(10.5)	35.4	(10.4)	0.28
	BP	33.5	(14.4)	34.0	(14.0)	33.5	(14.5)	33.1	(14.4)	0.54
	GH	32.1	(11.9)	33.5	(12.0)	33.0	(11.5)	33.2	(11.6)	0.01
Mental	VT	42.2	(11.1)	42.2	(11.0)	42.6	(11.5)	41.8	(11.5)	0.24
	SF	33.5	(13.4)	34.3	(13.3)	34.1	(13.5)	33.2	(13.4)	0.19
	RE	32.9	(12.8)	34.1	(12.7)	33.3	(12.8)	33.6	(12.6)	0.17
	MH	37.5	(12.2)	38.6	(12.1)	38.4	(12.1)	38.0	(12.0)	0.10
Summary	PCS	34.5	(11.5)	35.0	(11.4)	34.9	(11.6)	34.6	(11.8)	0.17
	MCS	37.5	(12.5)	38.5	(12.1)	38.2	(12.2)	37.8	(10.4)	0.21

**Table 5:** Test-Retest Correlation Coefficients in Control Group (n = 417)

Variable	Pearson’s Correlation Coefficient between Two Years (95% CI)						Adjusted ICC^a^
	BL-Y1	BL -Y2	BL -Y3	Y1-Y2	Y1-Y3	Y2-Y3	
PF	0.63 (0.57,0.69)	0.66 (0.60,0.71)	0.57 (0.50,0.63)	0.67 (0.62,0.72)	0.60 (0.53,0.66)	0.62 (0.56,0.68)	0.54
RP	0.48 (0.40,0.55)	0.51 (0.43,0.57)	0.48 (0.40,0.55)	0.49 (0.41,0.56)	0.51 (0.43,0.58)	0.54 (0.47,0.61)	0.44
BP	0.57 (0.50,0.63)	0.60 (0.54,0.66)	0.52 (0.45,0.59)	0.58 (0.51,0.64)	0.51 (0.43,0.57)	0.57 (0.50,0.63)	0.48
GH	0.55 (0.48,0.61)	0.58 (0.51,0.64)	0.55 (0.48,0.61)	0.57 (0.50,0.63)	0.56 (0.50,0.63)	0.58 (0.51,0.64)	0.51
VT	0.47 (0.40,0.55)	0.47 (0.39,0.54)	0.46 (0.38,0.53)	0.48 (0.40,0.55)	0.46 (0.38,0.54)	0.52 (0.45,0.59)	0.45
SF	0.43 (0.35,0.51)	0.42 (0.34,0.49)	0.39 (0.30,0.47)	0.50 (0.42,0.57)	0.39 (0.31,0.47)	0.49 (0.41,0.56)	0.42
RE	0.44 (0.35,0.51)	0.48 (0.40,0.55)	0.50 (0.43,0.57)	0.52 (0.45,0.59)	0.45 (0.37,0.52)	0.47 (0.39,0.54)	0.43
MH	0.57 (0.50,0.63)	0.53 (0.46,0.60)	0.49 (0.41,0.56)	0.55 (0.48,0.61)	0.50 (0.42,0.57)	0.55 (0.47,0.61)	0.52
PCS	0.70 (0.64,0.74)	0.71 (0.66,0.76)	0.61 (0.55,0.67)	0.71 (0.65,0.75)	0.64 (0.58,0.70)	0.71 (0.66,0.75)	0.61
MCS	0.58 (0.51,0.64)	0.58 (0.52,0.64)	0.51 (0.44,0.58)	0.61 (0.54,0.67)	0.52 (0.45,0.59)	0.61 (0.55,0.67)	0.57

^a^ For each scale, the ICCs were computed using four repeated assessments (baseline, year 1, 2 and 3), adjusting for age, sex, race/ethnicity, and CRG

## Discussion

Although SF-12v2® reliability has been previously reported in a few other studies [9, 16], our study is the first to demonstrate the good reliability of SF-12v2® to assess HRQOL in a population with behavioral conditions or SMI whose conditions are severe enough to qualify for supplemental security income. Cheak-Zamora et al. reported the reliability in SF12v2® in a general, civilian, non-institutionalized population enrolled in the 2003–2004 Medical Expenditure Panel Survey. They reported that the Mosier’s alpha for internal consistency was 0.88 for PCS and 0.82 for MCS, which is higher than the Mosier’s alpha that we observed in patients with behavior conditions or SMI. Slight attenuation of the reliability in this population was expected since the participants are chronically ill and the response shift may cause ambiguous HRQOL measures [21].

Our study also provided the test-retest correlations of individual scales and summary scores of SF-12 across three years in a population with behavioral conditions or SMI. Since the WIN study was not originally designed to assess test-retest reliability of SF-12, the time interval that the retests were administered is one year, which is longer than the time interval used to assess the test-retest reliability of an instrument traditionally. We reported estimates of the long-term (annual) test-retest correlations, which include the effect of longitudinal decay in the reliability. These correlations may serve as a lower bound of the test-retest reliability of the instrument defined traditionally. These results can be used to estimate the covariance structure, an essential component in computing power or sample size for any longitudinal study using SF-12 as an outcome [22]. Previously, Cheak-Zamora et al. reported that the test-retest reliability for SF-12v2® one year apart was 0.78 for PCS and 0.60 for MCS [16] using the Medical Expenditure Panel Survey data, which is consistent with the test-retest correlations reported in our study. The mean SF-12 physical and mental scores in Table 4 are consistently lower (34.5–35.0 for PCS and 37.5–38.5 for MCS) than the scores for the general US population which is 50 ± 10 for both PCS and MCS, consistent with the physical and mental or behavioral illness of these participants. Our interpretation of the correlation between the annual assessments is that the middle-aged Medicaid enrollees who participated were clinically ill but relatively stable during the study period. The explanation is consistent with the fact that the mean scores for all the individual scales had minimal changes across three years, as shown in Table 4.

There are a few limitations in our study. First, the study cohort is a heterogeneous disease population due to the nature of this pragmatic trial, which may limit the generalizability of the results to a specific disease population. We adjusted the ICCs of the scales by CRG to account for the heterogeneity of the clinical conditions of the participants. Second, we did not assess the short-term test-retest reliability, for instance, within a few weeks. Salyers et al. previously computed test-retest reliability for patients with SMI within a week and reported ICC = 0.73 for PCS and 0.80 for MCS in SF-12v1 [9], which filled in the gap to a certain extent.

## Conclusions

Consistent with previously reported correlations in various populations, the SF-12v2® gives stable correlations in a previously unstudied Medicaid population with a combination of physical and behavioral conditions or SMI. The results encourage using the SF-12v2® to assess HRQOL in such cohorts with chronic health conditions. The reliabilities of individual scales as well as the summary scores of SF-12 can be used to estimate the variability and covariance structure of the measures when estimating power or sample size for future studies [22]. Moreover, the modestly attenuated correlations in participants with combined physical and mental or behavioral conditions compared to that in the general population need to be considered in future study planning.

## Abbreviations

BP: Body Pain
COPD: Chronic Obstructive Pulmonary Disease
CRG: Clinical risk group
GH: General Health
HRQOL: Health-Related Quality Of Life
ICC: Intraclass Correlation
MCS: Mental Composite Score
MH: Mental Health
MI: Motivational Interviewing
MOS: Medical Outcomes Study
PCS: Physical Composite Score
PF: Physical Functioning
RE: Role Emotional
RP: Role Physical
SDA: Service Delivery Area
SF: Social Functioning
SF-12: Short From SF-12
SF-36: 36-item Short-From Health Survey
SMI: Serious Mental Illness
VT: Vitality
WIN: Wellness Incentive and Navigation
